# 1449. Evaluation of Cloud-Based Automated Analytic Pipelines for Whole Genome Sequencing Analysis of Multidrug-Resistant Gram-Negative Organism Outbreaks

**DOI:** 10.1093/ofid/ofad500.1286

**Published:** 2023-11-27

**Authors:** Emily B Jacobs, Tracy Ross, Yehudit Bergman, Shawna Lewis, Karen C Carroll, Patricia J Simner

**Affiliations:** Johns Hopkins School of Medicine, Timonium, Maryland; The Johns Hopkins Hospital, Baltimore, Maryland; Johns Hopkins, Baltimore, Maryland; Johns Hopkins University, BALTIMORE, Maryland; Johns Hopkins University, BALTIMORE, Maryland; Johns Hopkins School of Medicine, Timonium, Maryland

## Abstract

**Background:**

Clinical implementation of whole genome sequencing (WGS) has been identified as the optimal approach to identify transmission of multidrug-resistant (MDR) organisms in the healthcare setting. Although WGS capabilities are becoming more common in clinical laboratories, analysis and interpretation of results is still a barrier to implementation. The purpose of this study was to evaluate cloud-based automated analytic pipelines to aid with WGS analysis of MDR gram-negative organisms as part of outbreak investigations.

**Methods:**

A convenience set of MDR *Enterobacterales* (n: 142), *Acinetobacter baumannii* complex (n: 14) and *Pseudomonas aeruginosa* (n:29) isolates were WGS on Illumina sequencing platforms. The FASTQ files were uploaded to AREScloud (Ares Genetics, an OpGen group company) and to EPISEQ CS (bioMérieux) for whole genome or core genome multi-locus sequence typing (wgMLST/cgMLST) with dendrogram creation. Results were compared to traditional 7 gene MLST schema. Ease-of-use and additional tools were compared between pipelines.

**Results:**

AREScloud and EPISEQ CS generated similar dendrograms for all organisms evaluated (examples provided in Figures 1 & 2). EPISEQ CS provided preset similarity thresholds based on percent relatedness to define clusters simplifying outbreak analysis, whereas AREScloud allowed users to define allelic difference cutoffs. Although the wgMLST/cgMLST clusters aligned with traditional MLST types, not all isolates assigned to a similar MLST were identified as part of a related cluster demonstrating increased resolution of wgMLST and cgMLST phylogenetic assessments. Table 1 compares the features between the two pipelines.

Dendrogram generated for Escherichia coli (EPISEQ CS)

**Figure:**
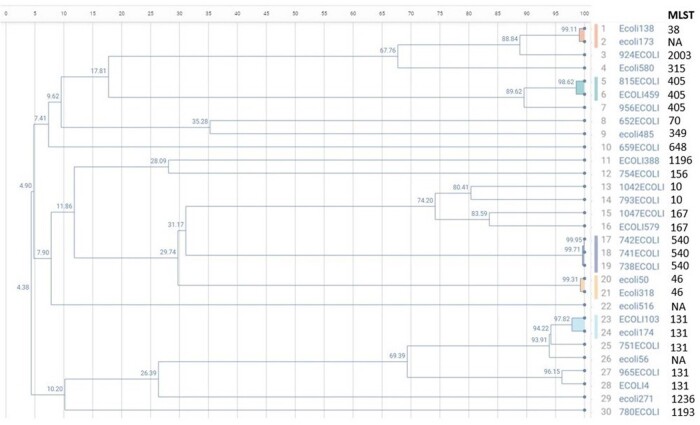

Dendrogram generated for Escherichia coli (AREScloud)

**Figure:**
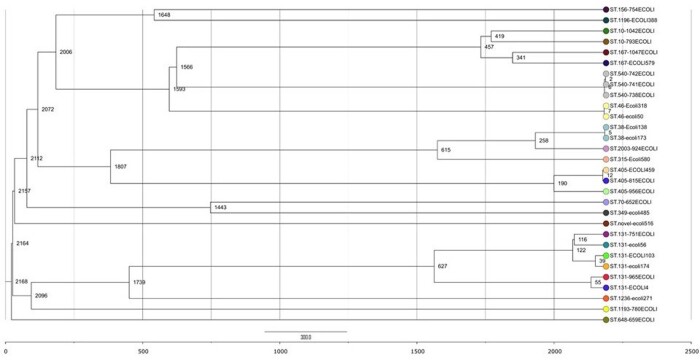

Comparison of features between EPISEQ CS and AREScloud for phylogenetic analysis

**Figure:**
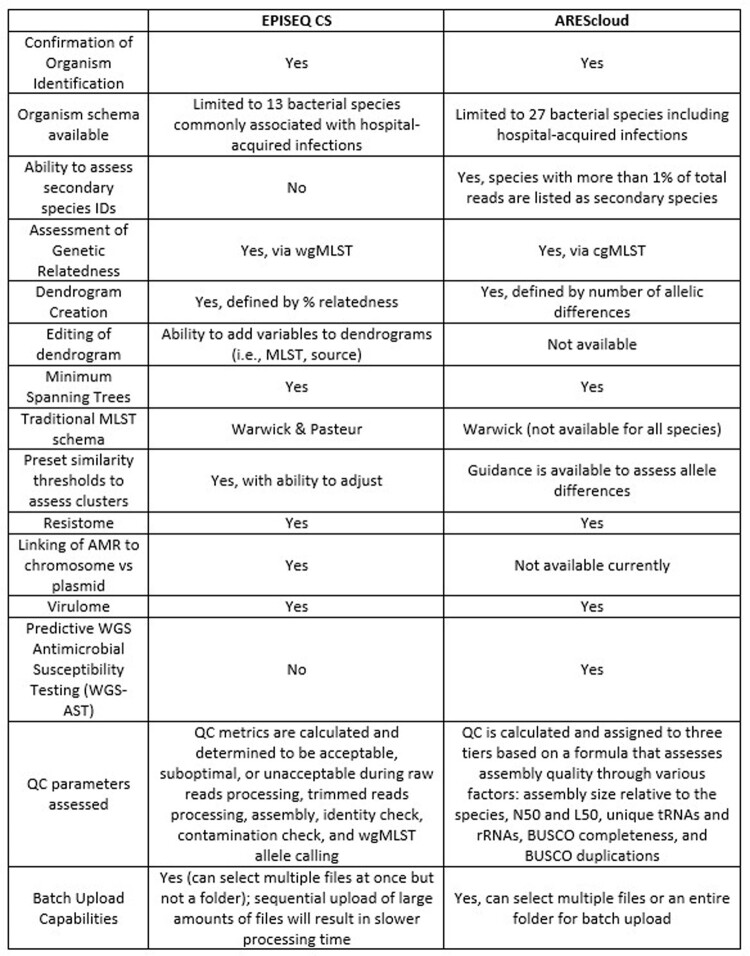

**Conclusion:**

Both cloud-based tools perform comparably for assessing phylogenetic relatedness compared to traditional MLST. Preset similarity thresholds available with EPISEQ CS provide ease-of-use for cluster assessment. Additional features, such as resistome and virulome analysis, are available by both pipelines. Predictive WGS-AST is an additional asset available with AREScloud.

**Disclosures:**

**Karen C. Carroll, MD**, Abbott Diagnostics: Board Member|Co-Diagnostics, Inc.: Board Member|Great Basin: Grant/Research Support|Meridian Diagnostics: Grant/Research Support|Pattern Diagnostics: Advisor/Consultant|Pattern Diagnostics: Stocks/Bonds|Qiagen, Inc.: Grant/Research Support|Scanogen: Advisor/Consultant|Scanogen: Grant/Research Support **Patricia J. Simner, PhD**, Affinity Biosensors: Grant/Research Support|BD Diagnostics: Advisor/Consultant|BD Diagnostics: Grant/Research Support|Entasis: Advisor/Consultant|GeneCapture: Stocks/Bonds|Merck: Advisor/Consultant|OpGen Inc: Board Member|OpGen Inc: Grant/Research Support|OpGen Inc: Honoraria|Qiagen Sciences Inc: Advisor/Consultant|Qiagen Sciences Inc: Grant/Research Support|Shionogi Inc: Advisor/Consultant|T2 Biosystems: Grant/Research Support

